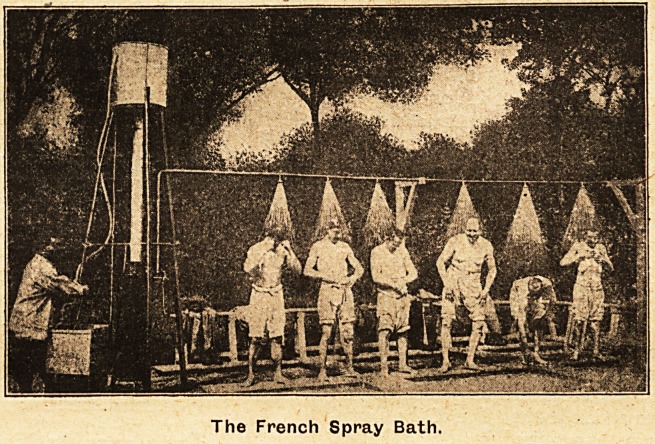# The Great War: X. Personal Cleanliness

**Published:** 1918-07-13

**Authors:** 


### THE GREAT WAR.

### X. PERSONAL CLEANLINESS.

### Vermin.

No soldier on the fighting front has yet been kept entirely free from vermin. The *Pediculus corporis* is universally prevalent, and has defied all attempts to defeat it.

The conditions of life on crowded battle fronts favour the spread of vermin. A constantly changing population of millions, occupying dug-outs, and billets in cellars, stables, and barns, all huddled together shoulder to shoulder, offers a continuity of humanity and a choice of food which is irresistible to the wandering parasite. The requisite personal cleanliness for those holding the line or engaged in active operations is impracticable; an army soon becomes infested, and every article of clothing and every blanket soon harbours eggs, which, owing to inevitable lapses in administrative routine, persist as perennial foci of dissemination.

LIFE HISTORY.

The female pediculus reaches maturity in from ten to fourteen days, and commences to deposit eggs a few days afterwards; from ten to twelve eggs are laid daily, and as the duration of life is from forty to fifty days, each female may lay several hundreds. Lice which are parted from their host die in about a week, and if deprived of their diet of blood, egg production ceases.

The favourite sites of oviposition are the seams of woollen clothing where it lies in closest contact with the body; *e.g.*, in the neck and the armpits, and in uniform jackets and trousers, in the seams of the collar and of the legs; the string suspending the identity discs around the neck is also a favourite site, and as this is usually overlooked in the scheme of disinfection it is a frequent source of infection.

In clothing which is constantly worn eggs hatch out in from seven to ten days, sooner or later, according to the temperature. In clothing which is not worn constantly hatching is indefinitely retarded.

PREVENTIVE MEASURES.

The general scheme of prevention is a weekly bath and change of underclothing and the destruction of eggs in the seams of uniform jackets and trousers by hot-ironing; blankets, when they are in use, are also constantly disinfected in steam or sulphur disinfectors. The application of these measures may appear to be very simple, but when millions in constant movement are concerned it becomes a vast undertaking requiring most meticulous organisation.

The infantry holding the line are most deeply concerned in the weekly bath; their difficulty in obtaining it is often insurmountable. The military situation is apt to interrupt the usual reliefs, and baths in the trenches are not popular for evident reasons.

Other branches of the Service do not fare so badly; they are further removed from rifle, bomb, and trench-mortar fire, and are able to make arrangements for interim baths locally.

The French Spray Bath.

THE FRENCH SPRAY BATH.

For simplicity, portability, efficiency, and economy of water, fuel, and labour the hot spray bath stands pre-eminently at the head of all systems of bathing for large numbers.

The British Army is under a great obligation to the French for the invention of their excellent pattern portable spray bath which we show on this page; it was distributed to the French Army in the early days of the war by the "Coordination des secours volontaires en faveur des soldats," whose office is at 57 Rue Saint-Dominique, Paris. It consists of a small circulating boiler, an elevated hot-water tank, a cold-water feeding tank, and metal pipes to which "roses" are attached at intervals of about three feet. The whole apparatus weighs only three cwt. and is packed in four compact wooden cases; it can be unpacked and fixed ready for use in an hour or taken down and packed for transport in the same time.

It is made in four sizes—four, six, and eight sprays—and sold at 425, 500, and 600 francs respectively.

It is most economical in working—40 to 50 lbs. of coal only are required *per diem* according to the size of the apparatus, and ½ gallon of water

Previous articles appeared Feb. 9, 23, March 9, 23, April 6, 20, May 4, 25, June 8 and 22, p. 247.

**The Great War—(*continued*).**

per spray per minute (2 gallons for each man), which is delivered at a uniform temperature of 40° C.; the water feed is by rotary pump which can be worked by hand, or by electric or petrol engine power.

This apparatus was introduced to the British Army on the initiative of an enterprising Medical Officer on the Headquarters Staff of the 8th Division in 1915, and its reputation slowly and gradually spread until it was officially adopted, and finally improved upon by British makers.

### Baths for Tommies.

The gratitude of the Army is also due to the society of ladies known as "Baths for Tommies," which provides complete bathing outfits, consisting of a boiler and a nest of three or four oval tin baths, in which it is possible to have a luxurious wash. They are to be found in most of the Field Ambulances and in many of the small units.

### The Bathing Parade at the Front.

The resting areas behind the whole of the British Front are dotted with baths conveniently situated for the access of the troops; they are located in schools, seminaries, coal-mines, breweries, distilleries, and in margarine and other factories, and in the devastated areas, in specially constructed huts. In many of the mines and factories spare hot water or steam blast is available and is used in tubs; when no hot water is available the prevailing system is the spray bath.

Divisions were formerly responsible for finding bathing accommodation for themselves as they moved about from place to place. All baths are now, fortunately, under the administration of Town-Majors in the towns and of Area Commandants in the devastated areas, an improvement which has revolutionised the bath problem. Bathing parades are carried out under Brigade arrangements; all that is necessary is a visit to the office of the Town-Major to book the baths required for the battalions. The men are then marched down on the appointed day, generally in platoons, at regular intervals.

Baths vary in capacity, the commonest types are eight-spray (French), twelve-spray (R.E.), and tubs varying from twenty to sixty in number, the number required being dependent on the labour available for preparing them. They are filled by buckets from large tanks heated by blast or from large boilers, a slow and tedious process.

We will describe the procedure at a double eight-spray establishment which is capable of bathing a battalion daily, at the rate of ninety-six men per hour. The first party of sixteen men enters an undressing-room for outer garments, where their greatcoats, boots, and puttees are numbered and stored; they then proceed to an undressing-room (A). Here they strip, turn their jackets and trousers inside out, and enter the baths, while orderlies pass their dirty underclothing through one window and their jackets and trousers, duly ticketed, through another window to the ironing-room.

As soon as the whistle sounds as a sign for the "A" party to enter the baths, another party enter a second dressing-room, "B," and prepare to follow the "A" party when they leave the baths. This they do at the sound of the whistle at the end of six minutes and proceed to the drying-room, where towels are provided. The "B" party take their places in the bathroom at the same time and follow the same routine.

When dried, "A" party proceed to their dressing-room and dress, having drawn clean underclothing from the store on the way; their jackets and trousers having been hot-ironed for the destruction of lice-eggs, are placed on the pegs, corresponding to the numbered tickets attached to them.

The destruction of lice-eggs on khaki clothing is a very essential link in preventive procedure; treatment by a *properly heated* iron is the most practicable remedy; the various anti-verminous pastes and powders which have been experimented with have proved failures. Cresol emulsion 5 per cent. to 10 per cent. in 50 per cent. of soft soap is the best, but its action is slow and does not prevent feeding until in full action.

Naphthalene is the most efficient quick-acting remedy, but its action is evanescent; it was tried very thoroughly in the German Army and was found inefficient.

For the efficient disinfection of the jackets and trousers of 100 men per hour, a separate establishment on the following scale is essential: Ironers, 8 (these are women where practicable); irons, 16; stoves for irons, 2. The very common custom of utilising the labour of waiting men for the ironing process has proved a failure, experience in the heating and the application of the iron is essential to success. A permanent and reliable superintendent is also indispensable.

(*To be continued.*)

## Figures and Tables

**Figure f1:**